# P-1564. Clinical Findings in Pediatric Patients with Renal Abscesses Treated with Intravenous versus Oral Antibiotics

**DOI:** 10.1093/ofid/ofae631.1731

**Published:** 2025-01-29

**Authors:** Kristine A Jermakian, Jared Olson, Jena Stallsmith, Sonia Mehra, Emily A Thorell, Timothy Benvegnu, Roger Wilcox

**Affiliations:** Primary Children's Hospital, Salt Lake City, Utah; Primary Children's Hospital, Salt Lake City, Utah; Primary Children's Medical Center, Salt Lake City, Utah; University of Utah, Salt Lake City, UT; University of Utah, Salt Lake City, UT; Primary Children's Hospital, Salt Lake City, Utah; Primary Children's Hospital - Salt Lake City, Salt Lake City, Utah

## Abstract

**Background:**

Renal abscesses in pediatric patients are uncommon and there is a paucity of data published regarding clinical findings and treatment outcomes. Case reports have documented the successful use of intravenous (IV) antibiotics, with or without surgical intervention. However, few case series have documented the use of enteral or oral antibiotics, specifically in the pediatric population. The treatment approach to renal abscesses in pediatric patients remains controversial.

**Methods:**

This retrospective case study identified children less than or equal to 18 years of age in the Intermountain Health System who had a radiographically diagnosed renal or perirenal abscess between 2015 and 2023. Patient charts were retrospectively reviewed and evaluated for clinical variables and treatment strategies. Patient and clinical outcomes assessed included hospital readmission, emergency department (ED) revisits, re-occurrence of abscess within one year, and length of hospital stay.

**Results:**

Forty-two patients were evaluated. On average, patients included had an elevated C-reactive protein and serum white blood cell (WBC) count. *Escherichia coli* was the main pathogen isolated from culture results (50%). Per urinalysis (UA) findings, 50% of UAs had trace or greater leukocyte esterase and 61% had trace or greater protein. Hematuria was observed in 60% of microscopic UA results, and 66% of UAs had WBCs greater than five. The majority of patients (81%) were sent home on oral antibiotics. Of the patients sent home on IV antibiotics, 71% of patients had an oral option. Readmission within 90-days was seen in 6% of patients on oral therapy and 14% of patients on IV therapy (P = 0.48). ED revisit within 90-days was seen in 6% of patients on oral therapy versus 14% of patients on IV therapy (P = 1.00). No patients had a re-occurrence of abscess within one year (P = 1.00). The median length of stay in the oral therapy group and IV therapy group was 3 days (2, 5.75) and 5.5 days (3.5, 8.75) (P = 0.13), respectively.

**Conclusion:**

No patients had a re-occurrence of abscess within one year. 71% of patients treated with IV antibiotics had an oral option and were treated with an IV antibiotic based on provider preference. Oral antibiotics appear to be effective in the treatment of renal abscesses.

**Disclosures:**

**All Authors**: No reported disclosures

